# The impact of expanded access programs for systemic anticancer therapy in an Irish cancer centre

**DOI:** 10.1007/s11845-024-03701-w

**Published:** 2024-05-28

**Authors:** Timothy K. Cronin, Cian Ronayne, Niamh O’Donovan, Eimear McGuinness, Katie Cooke, Maeve Dennehy, Colum Dennehy, Derek G. Power, Mary R. Cahill, Dearbhaile C. Collins, Roisin M. Connolly, Richard M. Bambury, Vitaliy Mykytiv, Michaela J. Higgins, Sinéad A. Noonan, Seamus O’Reilly

**Affiliations:** 1 School of Medicine and Health, University College Cork, Cork, Ireland; 2 Cork University Hospital, Wilton, Cork Ireland; 3 South Infirmary Victoria University Hospital, Old Blackrock Road, Cork, Ireland; 4 Mercy University Hospital, Grenville Place, Cork, Ireland; 5 Cancer Research @ UCC, University College Cork, Cork, Ireland; 6 St Vincent’s University Hospital, Elm Park, Dublin, Ireland

**Keywords:** Cancer, Compassionate use, Expanded access programs, Ireland

## Abstract

**Background:**

Expanded access programs (EAPs) allow cancer patients with unmet clinical need to obtain access to pre-authorisation treatments. There is no standardised process for implementing these programs nationally, and real-world data on their impact is lacking.

**Aims:**

This study aimed to evaluate the prevalence of such EAPs and their impact in a cancer centre.

**Methods:**

Data relating to adult cancer patients treated via EAPs from 2011 to 2021 in three Cork university hospitals was collated. Descriptive statistics were employed to get an overview of the impact these programs currently have on cancer care provision.

**Results:**

We identified 193 patients who accessed EAPs during the study period, availing of 33 separate drugs for a total of 50 different cancer indications. The prevalence of EAP usage was shown to have been trending upwards in recent years with a total of 189 programs being accessed throughout the period. Drugs provided were from a number of different anti-cancer drug classes, particularly targeted therapies (*n* = 18) and immune checkpoint inhibitors (*n* = 17). Cancers from a wide range of both solid and liquid tumour types were treated with EAP drugs, and patients treated were from across a broad spectrum of ages (26–82, SD 11.99).

**Conclusions:**

EAPs have an increasing role in accessing novel cancer therapies in our community and by extension nationally. Equity of EAP access would be facilitated by a national registry of available agents which we have established. Assessment of their benefits and toxicities would be enhanced by the requirement for a real-world database as a condition of EAP approval.

## Introduction

Expanded access describes the therapeutic use of investigational drugs outside of clinical trials [1]. Other terms such as ‘compassionate use’ and ‘early access’ have been used, often synonymously, to describe this use of unlicensed drugs prior to formal regulatory licensing decisions [2–4]. Patients availing of such programs include those for whom the drug is believed may have clinical benefit but who are otherwise unable to access treatment, for example due to failing to meet eligibility criteria for clinical trials, or when clinical trials are no longer accepting patients, or when the agent is approved by regulatory bodies but not yet reimbursed by the state or third parties [2].

The regulatory approach to such expanded access programs (EAP) varies widely amongst jurisdictions. For instance in the United States (US), the Food and Drug Administration (FDA) provides a formal definition of expanded access use [5], whereas in Europe, the European Medicine Agency (EMA) has a much broader definition, similar to that of an open label extension study and early access to investigational drugs is governed and operated through various mechanisms specific to individual member states [6]. Notably in the US, the Right to Try Act, which became law in 2018, presents another avenue to early therapeutic use of experimental or investigational drugs [7, 8]. Therefore, with little concordance between regulatory bodies about what expanded access entails, patients are forced to navigate a disconnected network of individual drug programs in order to gain access to potentially beneficial treatments. With such heterogeneity between various programs as well as between individual countries, extensive research into EAPs is lacking. As a result, information and awareness of these programs have been shown to be poor, even amongst experienced physicians who are responsible for availing of them in the treatment of their patients [8].

In Ireland unlike most other European countries, there is no national protocol or standardised approach for the implementation of EAPs [9]. Consequently, there is a gap in the literature in terms of data and research examining the role that these programs play in current cancer patient care in Ireland. Equally, lack of transparency or a national database compromises equity of access to patients nor is there real-world data on safety and efficacy. The aim of this study was to examine the extent of usage of EAPs in three Irish hospitals including Cork University Hospital (CUH), the Mercy University Hospital (MUH), and the South Infirmary Victoria University Hospital (SIVUH) to assess the impact of EAPs on cancer care.

## Methods

This was a retrospective study pertaining to patients granted access to cancer treatment via EAPs in three university hospitals in the Southwest of Ireland from January 2011 to December 2021. EAPs were identified by treating physicians. All of the accessed programs were administered through hospital pharmacies only. Enrolled patients were identified using hospital pharmacy data and the delivery of available agents was provided through hospital pharmacies only. Treatment records were assessed using paper and electronic sources. Descriptive statistics were used to analyse the data. Data collection took place from September 2021 to June 2022 inclusively. Data analysis took place between June 2022 and October 2022. Data was collated and analysed using Microsoft® Excel® (Version 2210).

Ethical approval was granted from the Clinical Research Ethics Committee of the Cork Teaching Hospitals (CREC) in July 2021.

## Results

In total, 205 patients were identified as having been granted approval for access to treatment via an EAP during the study period. The median age at the time of original cancer diagnosis pertaining to the indication for EAP treatment in studied patients was 73 (range 26–82, SD 11.99). Of these patients, 115 (56.10%) were women and 90 (43.90%) were men. Fifteen patients did not start treatment either due to clinical deterioration which deemed them unfit for further treatment, or death in the interim between program approval and treatment initiation. Of the remaining 193 patients identified, patients were treated across a wide spectrum of ages, with a median age at initiation of an EAP of 74.5 (30–83, SD 11.28).

Treatments were provided from across a range of 33 total drugs during the study period. These are outlined in Table 1 below along with the respective numbers of patients treated at each clinical hospital site according to drug.

**Table 1:** Drugs used in respective hospitals by patient number

Drug		Number of patients treated		
	CUH		MUH	SIVUH
Alemtuzumab	1			
Alpelisib				2
Amivantamab	1			
Apalutamide			2	
Atezolizumab	19			
Avelumab	1		1	
Belantamab mafodotin	2			
Brentuximab vedotin	1			
Cabozantinib	3			
Cemiplimab	1			
Durvalumab	9		2	
Erdafitinib	1			
Gemtuzumab ozogamicin	6			
Gilteritinib	3			
Inotuzumab ozogamicin	1			
Ipilimumab and Nivolumab	3		2	
Ixazomib	3			
Neratinib	4			7
Nivolumab	14		27	2
Olaparib	2			
Osimertinib	2			
Palbociclib	6			7
Pembrolizumab	8		18	
Polatuzumab vedotin	1			
Quizartinib	1			
Regorafenib				1
Ribociclib	5			1
Rucaparib	14			2
Selinexor	2			
Sotorasib	1			
Talazoparib	1			
Venetoclax	2			
Vismodegib	1			

**Fig. 1:**
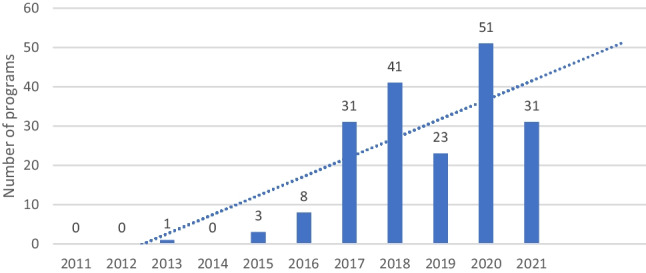
Number of EAPs implemented by year

The earliest recorded EAP treatment was administered in July 2013. Over the following years, the number of individual programs administered followed a general upward trend as shown in Fig. 1 above (*R*^2^ = 0.731).

**Fig. 2:**
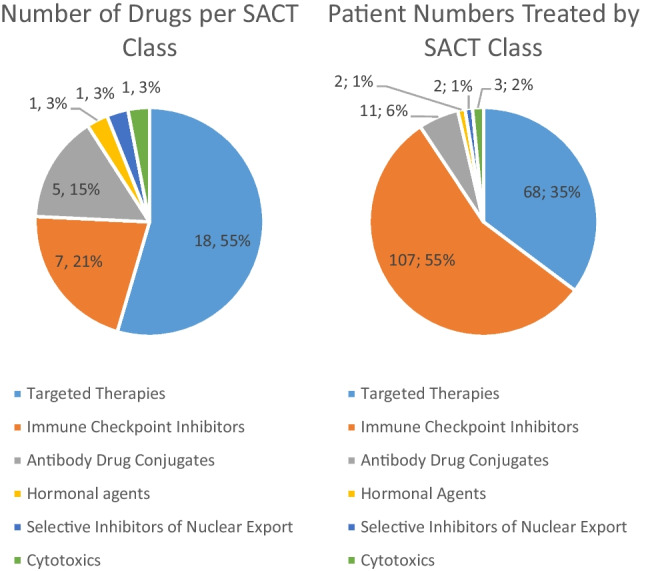
EAP SACT drugs provided by drug class and patient numbers by respective SACT class

The drugs in the studied EAPs came from multiple systemic anticancer treatment (SACT) drug classes as highlighted in Fig. 2. As shown, targeted therapies had the largest representation in terms of drug class in the EAPs included (*n* = 18, 55%), followed by immune checkpoint inhibitors (*n* = 7, 21%).

Notably, the largest number of patients were treated with an immune checkpoint inhibitor (*n* = 107, 55%) followed by targeted therapies (*n* = 68, 35%), accounting for 90% of patients compositely. The next largest group was the antibody drug conjugate patient group (*n* = 11, 6%), while the remaining patients (*n* = 7, 5%) were treated with drugs from the cytotoxic, hormonal agent and selective inhibitor of nuclear export groups. This is illustrated further in Fig. 2.

**Fig. 3:**
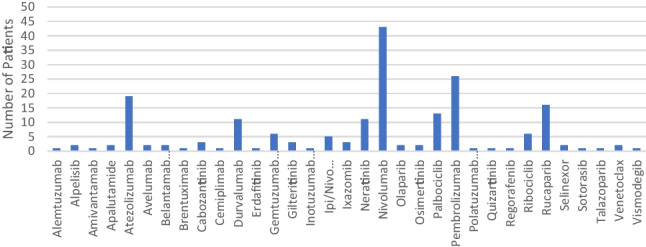
Number of patients treated with individual drugs

Most drugs were provided for a single indication, but in a number of cases, the drug was used for several indications through separate EAPs (Fig. 3). Nivolumab, an immune checkpoint inhibitor was used as monotherapy to treat 12 separate cancer indications and as dual therapy in addition to ipilimumab for a further indication. Detailed individual drug indications are described in Table 3.

Patients from across several cancer types were treated with individual EAPs over the course of the last 9 years. Lung cancer was the most common cancer type for which patients were treated under an EAP (*n* = 55), followed by breast cancer (*n* = 33), and ovarian cancer (*n* = 18). Further details regarding numbers of patients treated by cancer type are shown in Table 2.

**Table 2:** Number of patients treated by cancer type

Cancer type	Patient number
Lung	55
Breast	33
Ovarian	18
Gastroesophageal	16
Renal	14
Leukaemia	14
Urothelial	10
Multiple myeloma	7
Colorectal	7
Skin	6
Prostate	4
Lymphoma	4
Cervical	1
Endometrial	1
Head and neck	1
Brain	1
Cancer of unknown primary	1

The duration of treatment for each of these drugs according to respective indication is given below, as summarised in Table 3. In certain circumstances, only a single dose was administered, and in these instances, a treatment duration of 0 was assigned.

**Table 3:** Median durations of treatment for respective drugs by indication

Drug class	Drug	Indication/cancer type	Patient number	Median duration of treatment (weeks) (range, if applicable)
Targeted therapies	Alemtuzumab	T cell prolymphocytic leukaemia	1	9
	Alpelisib	Metastatic invasive ductal breast cancer	2	20 (0–40)
	Amivantamab	Metastatic lung adenocarcinoma	1	6
	Cabozantinib	Metastatic clear cell renal cell carcinoma	3	61 (9–161)
	Erdafitinib	Metastatic urothelial cell carcinoma	1	9
	Gilteritinib	Acute myeloid leukaemia	3	10 (7–22)
	Neratinib	Metastatic HER2 + invasive ductal breast cancer	11	33 (0–51)^a^
	Olaparib	Metastatic serous ovarian cancer	2	317 (300–334)
	Osimertinib	Metastatic lung adenocarcinoma	2	51.5 (9–94)
	Palbociclib	Breast cancer	13	37.5 (7–108)^b^
	Quizartinib	Acute myeloid leukaemia	1	21
	Regorafenib	Colorectal cancer	1	4
	Ribociclib	Metastatic breast cancer	6	18.5 (0–95)
	Rucaparib	Ovarian cancer	14	23 (0–184)
		Prostate cancer	2	76.5 (76–77)
	Sotorasib	Lung adenocarcinoma	1	2
	Talazoparib	Metastatic invasive ductal breast carcinoma	1	7
	Venetoclax	Acute myeloid leukaemia	2	6.5 (6–7)
	Vismodegib	Basal cell skin carcinoma secondary to Gorlin syndrome	1	165
Immune checkpoint inhibitors	Atezolizumab	Small-cell lung cancer	6	15 (0–158)
		Non-small cell lung cancer	13	9 (0–111)
	Avelumab	Merkel cell carcinoma	2	79.5 (16–143)
	Cemiplimab	Metastatic squamous cell skin carcinoma	1	154
	Durvalumab	Small-cell lung cancer	2	43.5 (16–71)
		Non-small-cell lung cancer	9	27 (0–55)
	Ipilimumab and Nivolumab	Metastatic clear cell renal cell carcinoma	5	9 (0–15)
	Nivolumab	Colorectal cancer	7	67.5 (0–188)
		Renal cell carcinoma	6	24.5 (5–78)
		Metastatic squamous cell skin carcinoma	2	19 (0–38)
		Glioblastoma multiforme	1	8
		Ovarian cancer	2	2.5 (1–4)
		Endometrial cancer	1	4
		Gastro-oesophageal cancer	16	38.5 (4–91)
		Urothelial cell carcinoma	3	19 (0–51)
		Non-small-cell lung cancer	3	115 (36–129)
		Carcinoma of unknown primary	1	2
		Cervical small-cell carcinoma	1	2
		Head and neck cancer	1	25
	Pembrolizumab	Non-small-cell lung cancer	18	15 (0–73)
		Hodgkin’s lymphoma	2	34.5 (0–69)
		Urothelial cell carcinoma	6	18 (0–97)
Antibody drug conjugates	Belantamab mafodotin	Multiple myeloma	2	0
	Brentuximab vedotin	Anaplastic large-cell lymphoma	1	15
	Gemtuzumab ozogamicin	Acute myeloid leukaemia	6	11 (0–24)
	Inotuzumab ozogamicin	Pre-B-cell acute lymphoblastic Leukaemia	1	11
	Polatuzumab vedotin	Diffuse large B cell lymphoma	1	21
Hormonal agents	Apalutamide	Prostate adenocarcinoma	2	33 (24–42)
Selective inhibitors of nuclear export	Selinexor	Multiple myeloma	2	5 (4–6)
Cytotoxics	Ixazomib	Multiple myeloma	3	11 (10–124)

^a^Note that treatment was only planned for 1 year (51 weeks) as per adjuvant treatment protocol.

^b^Note that complete treatment duration data was not available for three patients in this group and the value recorded is reflective of the remaining patients only

**Fig. 4:**
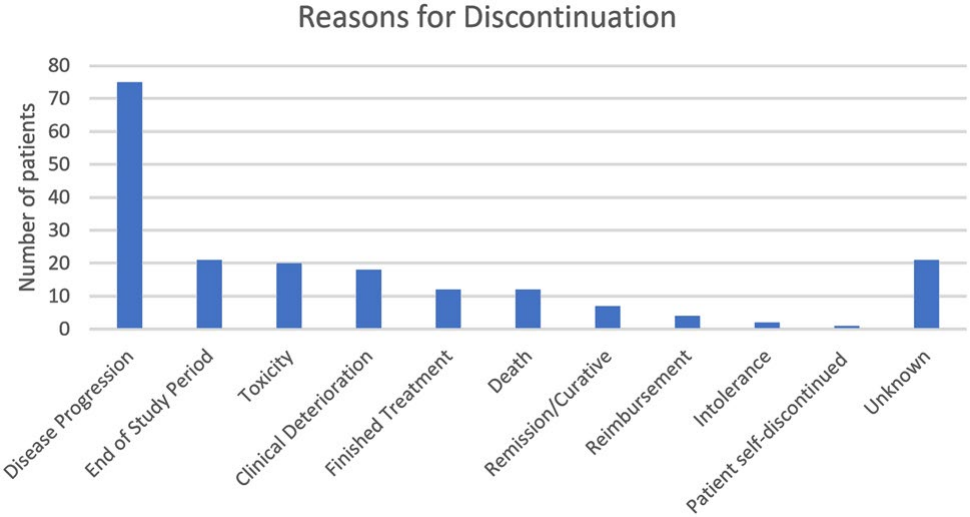
Reasons for cessation of EAP drug treatments by patient number

The main reason for discontinuation of EAP drug treatment was because of disease progression (*n* = 75, 38.86%). In the case of 21 patients (10.88%), the reason for treatment cessation was not known, either due to missing data or a lack of a formal recorded reason. In a further 21 patient cases (10.88%), treatment continued up to the last day of the data collection period and as such continued beyond the study’s timeframe. Notably, toxicity related to EAP treatment and intolerance to treatment combined were responsible for treatment discontinuation in 22 patients (11.39%). Remission and completion of the intended EAP treatment course combined were responsible for treatment discontinuation in 19 patients (9.84%). Figure 4 details the different reasons provided for treatment cessation.

## Discussion

The results of this study serve to illustrate the important role that EAPs have come to serve in Irish cancer treatment. EAPs provided otherwise unobtainable access to treatment for 193 patients over the past 9 years for a wide range of conditions and is the first study of its kind nationally. Importantly, the results also show that the number of EAPs being accessed per year increased in general year on year emphasising the increasing gap between drug approval and drug reimbursement in this jurisdiction. In total, 33 drugs were provided for a total of 50 different cancer indications reflecting the important role of EAPs in the landscape of cancer practice nationally.

During the time period assessed in this study, no central registry existed for EAPs. Their application was ad hoc and reflective of physician initiative and patient motivation. In the absence of a formal national registry, equity of access for patients is hard to assess. Provision of safety data from EAPs is voluntary and a condition of EAP provision. Compliance with reporting has not been formally assessed nationally in the time frame of the current study. A national register and data base would facilitate a clearer understanding of real-world experience and benefit of EAPs in this cohort. It would also remove the reliance on physicians’ individual awareness of available programs and streamline the application process. Such a national standardised protocol for EAP usage would also improve the regulation of these programs and bring Ireland in line with other European countries who have organised centralised processes.

Another specific consideration in Ireland’s case that may serve to further underscore the benefits of such a coordinated effort is the reimbursement process for anticancer treatments in Ireland. Recent work has shown that the median time between EMA drug approval and public reimbursement in Ireland is 18 months vs 9 months in the UK [10]. This lengthy interval between central European marketing authorisation and Irish public reimbursement puts a constraint on access for cancer patients to innovative treatments. In these cases, expanded access or compassionate use programs may serve to alleviate this burden, particularly if a patient has previously been granted access to such a program prior to licensing approval by the EMA. Therefore, with an organised and regulated approach, these patients may also derive benefit from treatment to which they may otherwise not have access. Co-operative research between treating physicians, pharmaceutical companies, and drug regulators in these cases may also help to ‘bridge the gap’ between regulatory decisions and payment and reimbursement decisions [11].

As mentioned, Ireland stands as an outlier in its lack of formalised approach to expanded access treatments in Europe [9]. In the UK for instance, there has been a national programme in place known as the Early Access to Medicines Scheme (EAMS), coordinated by the Medical and Healthcare products Regulatory Agency (MHRA) since April 2014 [12]. As a result of this scheme, a series of innovative drugs, primarily for oncological indications, have been granted positive Scientific Opinions allowing them to be brought to market approximately 12–18 months prior to marketing authorisation approval [13]. Since its establishment in April 2014 through to April 2022, the EAMS has seen 60 applications for a Scientific Opinion, of which 45 have been awarded a positive Scientific Opinion [14].

Importantly, each of these Scientific Opinions do not guarantee access for patients to agents designated as a Promising Innovative Medicine (PIM) according to step 1 of the EAMS process. Rather, should a patient be deemed suitable for such a treatment on a case-by-case basis, then the manufacturing pharmaceutical company may provide the treating physician with access to this PIM to treat the patient in question. This is similar in many ways to the current usage of EAPs in Ireland whereby requests by treating oncologists and haematologists are submitted on behalf of individual patients to pharmaceutical companies. These companies then determine on a case-by-case basis if these patients are suitable for early access to pre-approval medicines. The important difference however is that in the UK, this process is operated and coordinated in accordance with strict regulatory guidance and under the vigilance of several bodies including NICE, the NHS, and the MHRA. Similar approaches have been developed across Europe to handle early market access of such innovative medicines in a standardised fashion according to local guidelines, regulations, and patient needs [10].

In France, a system for expanded access has been in place since 1994 known as the ‘*Autorisation temporaire d’utilisation*’ (ATU), which allows for early access to investigational treatments in exceptional circumstances prior to a ‘marketing authorisation’ (MA) decisions by the EMA [15]. As part of this process, the product in question is subject to an authorisation for use granted by the National Agency of Medicine and Health Product Safety (Agence Nationale de Sécurité du Médicament et des Produits de Santé [ANSM]) on the basis of a supposed positive benefit/risk ratio [16]. Once granted this authorisation, pharmaceutical companies are free to determine the ATU price of their drug which is then reimbursed fully by the National Health Insurance in France [15, 16]. During the period between January 2007 and December 2019, 36 antineoplastic drugs were granted an ATU in France and as such were made available on average 428 days before EMA approval [15].

More recently, in the Netherlands, another approach has been developed for the expanded access use of investigational anticancer drugs known as the ‘DRUG Access Protocol’ (DAP) [17]. This protocol aims to coordinate on a national level, access to novel anticancer drugs while supporting research by obtaining additional data on drug safety and activity in a real-world setting [17]. Through this system, treatment is provided free of charge for up to 4 months by a pharmaceutical company, provided the relevant drug meets a predefined clinically meaningful benefit threshold. In the case of a demonstrated clinical benefit, treatment can be extended by up to a further 4 months which is reimbursed by relevant payers [17]. As described, many individual countries have developed their own systems and approaches to coordinating and providing expanding access cancer treatment which juxtaposes the current situation in Ireland. Given the extent to which these programs have now become an element of cancer treatment in recent years, this further accentuates the merits of a similarly coordinated and nationwide approach to their use in Ireland.

This study has several limitations. As it was a retrospective study, missing data was noted in several patients. The time from initiation of discussions about an EAP and patient receipt of treatment was not recorded nor was there recording of unsuccessful EAP applications. Safety data was in general lacking reflecting a lack of formalised collection. Finally, a prospective evaluation of patients’ satisfaction with EAP availability was not conducted nor was the impact of such treatments on quality of survival.

The descriptive nature of the statistical analysis used in this study limits the inferences that can be made as a result, particularly in gauging the true impact of EAPs on current cancer treatment clinical practice. Based on the results of the study, a national database of available EAPs has been established by the Irish Society of Medical Oncology. Members prospectively register newly available programs which facilitates equity of access to these programs nationally. The next needed step is the establishment of real-world data bases as *a proviso* for EAP establishment nationally with the development of a national registry.

## Data Availability

The anonymised datasets generated during and/or analysed during the current study are available from the corresponding author on reasonable request.
